# Impacts of feed gases for micro-nano bubble water treatments: Antimicrobial efficacy against *Escherichia coli* and *Staphylococcus aureus* on ‘Fan Retief’ guava fruit

**DOI:** 10.1007/s10068-025-01897-w

**Published:** 2025-05-20

**Authors:** Harold K. Malahlela, Zinash A. Belay, Rebogile R. Mphahlele, Lize Engelbrecht, Janica C. Theron, Oluwafemi J. Caleb

**Affiliations:** 1 Department of Food Science, Faculty of AgriSciences, Stellenbosch University, Matieland, South Africa; 2 AgriFood BioSystems and Technovation Research, Africa Institute for Postharvest Technology, Faculty of AgriSciences, Stellenbosch University, Matieland, South Africa; 3 Post-Harvest and Agro-Processing Technologies (PHATs), Agricultural Research Council (ARC) Infruitec-Nietvoorbij, Stellenbosch, South Africa; 4 Department of Land Reform and Rural Development, Private Bag X250, Pretoria, 0001 South Africa; 5 Central Analytical Facilities, Microscopy Unit, Stellenbosch University, Matieland, South Africa

**Keywords:** *Psidium guajava* L., Ozone, Microbiocidal, Micro-nano bubble, Confocal microscopy, Scanning transmission electron microscopy

## Abstract

**Abstract:**

Microbes on fresh produce are often controlled by chlorine-based sanitizers, due to there is growing demand for safe alternatives. This work investigated effects of micro-nano bubble (MNB) water generated using air, oxygen (O_2_), or ozone (O_3_) against bacteria contaminants encountered along the fresh produce value chain. *Escherichia coli* (ATCC 25922) and *Staphylococcus aureus* (ATCC 25923) broth were treated with distilled water (DW), sodium hypochlorite (NaOCl, 200 mg/L, 5 min), air-MNB, O_2_-MNB, and O_3_-MNB for 30- and 60-min. Scanning-transmission electron microscopy confirmed that the bacterial survival population was reduced via various types of cell damage under O_3_-MNB. *S. aureus* was more resistant than *E. coli* to the MNB treatments. This could be due to the thicker layer of peptidoglycan surrounding the cell membrane protecting against oxidative species. On guava fruits, O_3_-MNB-60 min lowered *E. coli* counts immediately after treatment (p < 0.05). MNB water offers a new paradigm for fruit decontamination.

**Graphical abstract:**

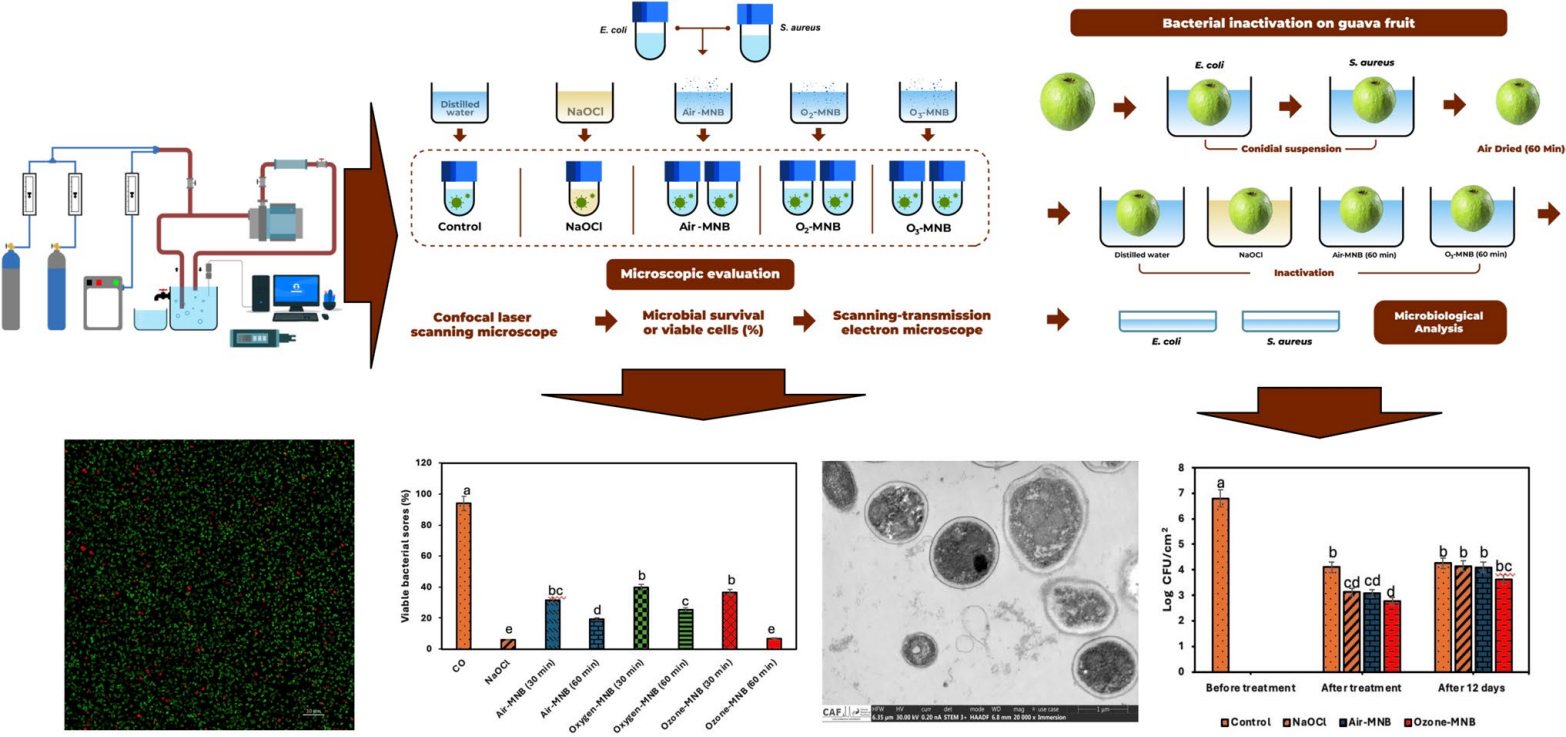

**Supplementary Information:**

The online version contains supplementary material available at 10.1007/s10068-025-01897-w.

## Introduction

Washing of fresh whole and/or minimally processed fruits and vegetables (FV) is crucial for decontaminating against foodborne pathogens and improving their appeal. These foodborne pathogens may originate from soil, manure, harvesting bins, irrigation waters, decay, or mechanical injuries (Mohanapriya et al., 2024). Over the years, the Food and Drug Administration documented 72 cases of illnesses linked to the intake of contaminated FV, which constitutes 25% of the total reported outbreaks from 1996 to 2006 (Food & Drug Administration, 2008). Although washing of FV is essential, this practice still pose a potential risk in transferring microbial pathogens to the washing waters, processing tools and equipment’s and transportation materials. Common traditional washing methods use chlorine-based cleaners (calcium and/or sodium hypochlorite) as agents to minimize risks of microbial cross-contamination.

Studies have showed that chlorine disinfectants can reduce 1–2 log CFU g/L of natural aerobic mespohillic bacteria, yeasts and molds on fresh cut zucchinis, cucumbers and green bell peppers (Sun et al., 2012). In addition, a 2.98 ± 0.19 log CFU/g reduction of *Salmonella enterica* was reported on iceberg lettuce washed with 25–80 mg/L NaOCl (Cuggino et al., 2023). However, there exists a significant decline in chlorine effectiveness within aqueous solutions due to oxidative properties which could negatively affect the overall quality of FV (Zhang & Tikekar, 2021). In addition, chlorine-based disinfectants are less effective in aqueous solutions with higher pH, lower temperatures and in hard water solutions. These conditions diminish the active disinfecting agent (hypochlorous acid), and favor of the less effective hypochlorite ion (Artasensi et al., 2021). Sodium hypochlorite is characterized by high instability with multiple parameters to manage for optimum efficacy. Thus, the exploration of other decontaminants is a crucial research area in elevating food safety standards in the FV industry.

Micro-nano bubble (MNB) technology offers a potential solution as a decontaminant. These MNB (1 μm or less gas cavities in diameter suspended in solution) have antimicrobial properties due to the production of free radicals (Agarwal et al., 2011). Their small nano-sized properties allow penetration into the microorganism’s cell walls, inactivating fungi and bacteria on FV surface. Moreover, the remarkable solubility and stability of MNB holds promise for their application in washing FV using various gases. One of the important gases used to generate MNB is O_3_. Ozone MNB has been investigated for its antimicrobial efficacy and impact on postharvest quality of FV (Inatsu et al., 2011; Hou et al., 2022; Saijai et al., 2019). These studies demonstrated that O_3_-MNB extends the postharvest life of FV by eliminating native microflora and oxidizing ethylene, and its efficacy in maintaining produce quality is notable. The recommended O_3_ concentration in water for FV disinfection varies depending on the type of produce, the desired level of sanitation and it ranges from 0.5 to 2.0 mg/L (Wang et al., 2019). Furthermore, the potential antimicrobial impact of O_3_-MNB generated water have been researched on spinach, (Xueqing et al., 2020), sweet basil (Phaephiphat and Mahakarnchanakul, 2018) and Chinese cabbage (Ushida et al., 2017). Zhang & Tikekar (2021) investigated the effect of air-MNB for microbial detachment and inactivation on tomato. Moreover, Shiroodi et al. (2021) investigated the efficacy of O_2_-MNB in removing *Vibrio parahaemolyticus* biofilms. Ahmed et al. (2018) studied seed and plant development focusing on the influence of MNB feed gases (air, CO_2_, N, and O_2_). However, there are currently no articles elucidating the impact of exposure to feed gases such as air-, O_2_-MNB and O_3_-MNB on cell viability (%) and changes in cellular structures for fresh produce cross-contaminants such as *E. coli* and *S. aureus* post-treatment.

Therefore, this work explores the impact of different feed gases in MNB (air-, O_2_-MNB and O_3_-MNB) as alternative decontaminant for packhouse management. This study illustrates: (a) the efficacy of air, O_2_- and O_3_-MNB water in comparison with the commercial NaOCl against known fresh produce bacterial cross-contaminants (*Escherichia coli* (ATCC 25922) and *Staphylococcus aureus* (ATCC 25923)) in cell suspension and on guava fruit surface; and (b) the underling mechanism of action for MNB water treatment against *E. coli* and *S. aureus* by examining changes in membrane and intracellular structures.

## Materials and methods

### Generation of micro-nano bubble water

**Fig. 1:**
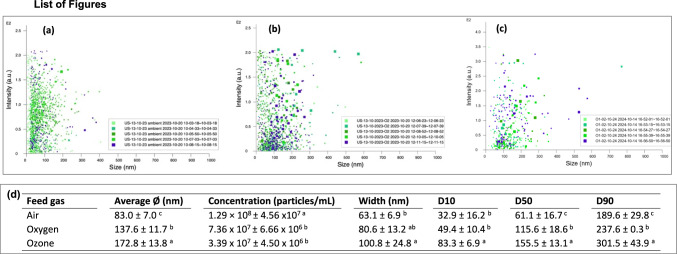
Scatter plot for size vs intensity of (**A**) air, (**B**) oxygen, (**C**) ozone micro-nano bubble water, and (**D**) the summarized report of size distribution profile and concentration of micro-nano bubble water with different feed gases. Results represent mean (*n* = 5) ± standard deviation; D10, D50 and D90 represents % percentage of micro-nano bubbles under sizes of 10, 50 and 90%, respectively. Similar lower-case letters along the columns are not significantly different at p ≤ 0.05

A MNB generator that produces ultra-fine bubbles (Ø ≈ 76.4 nm; concentration ≈ 2.22 × 10^10^ MNB/mL of water) (Mk4-NanoBubbler, Fine bubble technologies Co., Ltd., South Africa). The picture of the MNB generator system which utilized 3 different feed gases (air, O_2_ and O_3_) is shown in Supplementary (S)-Fig. 1. The MNB generator was connected to gas cylinder of O_2_ and fed into harvested rainwater (after multi-step microfiltration and ultrafiltration), which was recirculated through the pump to generate O_2_-MNB. For production of O_3_-MNB, an ozone generator was conneceted to the micro-nano bubbler to circulate O_3_ gas into the rainwater.

To produce air-MNB, the Mk4-Nanobubbler gas inlet tube was allowed to circulate air into fresh rainwater to generate air-MNB. The Mk4-Nanobubbler was allowed to recirculate the feed gases for 2 h to generate air, O_2_ and O_3_ MNB. After generation, the pH, oxidation–reduction potential (ORP) and temperature were measured using dual pH and ORP meter (HI 98121, Hanna Instruments, Cape Town, South Africa). As shown in Supplementary Table 1, DW showed a near neutral pH (6.4 ± 0.45). In contrast, commercial sanitizer NaOCl had an alkaline pH (10.7 ± 0.00), whereas air and O_2_ MNB had a slightly alkaline pH of 9.5 ± 0.50 and 9.3 ± 0.68, respectively. The pH value of O_3_-MNB was significantly lower (p ≤ 0.05), and acidic after 3 h of generation, and the lower pH was consistent with a higher ORP (919 ± 12.28 mV). On the other hand, O_2_-MNB and air-MNB had a lower ORP of 232 ± 12.00 and 188 ± 18.00 mV, respectively. In addition, the ORP of DW was 286 ± 31.00 mV and NaOCl showed a slightly higher ORP of 478 ± 1.00. All water samples were in a cool temperature range (18.1 ± 1.90–21.3 ± 0.30 °C), which implies that the treatments were non-thermal.

The bubble size distribution and concentration of MNB waters were monitored using the Malvern Panalytical NanoSight NS300 (Malvern Instruments Ltd., Worcestershire, UK), which is fully programmed via the Nano Tracking Analyzer (NTA) software v3.44 (Malvern Panalytical Ltd., Worcestershire, UK) with the average of five replicates per MNB type. The system is a laser-based and light scattering unit that detects particles in liquid samples moving under Brownian motion, whereby, MNB can be visualized in real time while generating nano-sight reports on the particle properties. The NanoSight reports were generated from 5 independent readings (*n* = 5).

### Microbial culture and inoculum preparation

*E. coli* (ATCC 25922) and *S. aureus* (ATCC 25923) Kwik-stik’s were purchased from Analytical Technology (Pty Ltd), Cape Town, South Africa. The kwik-stik’s of each bacterium were streaked on plate count agar (PCA) according to manufactures procedure, and kept for 48 h at 37 °C. Thereafter, a single colony from each strain working culture of was transferred into a 10 mL tube of nutrient broth and kept at 37 °C overnight. The cultures were collected and washed once via centrifugation for 10 min at 10,000×*g*, to obtain cell pellets. These pellets were the re-constituted to approximately 10^8^ CFU mL^−1^ in sterile distilled water using Ultravoilet–visible spectrometery absorbance of 0.15 at 600 nm (Thermo Scientific Technologies, Madison, Wisconsin), and the suspension was used for microbial decontamination.

### In-vitro bacterial inactivation test of micro-nano bubble water

**Fig. 2:**
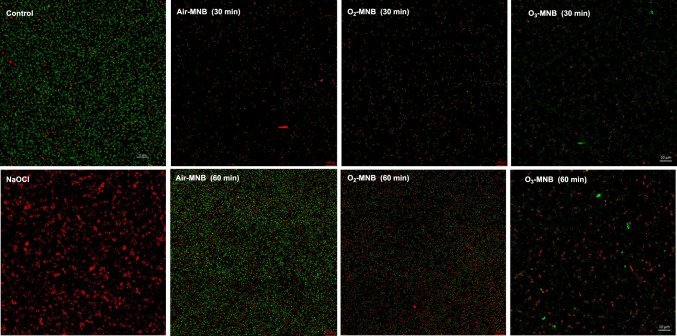
Confocal laser scanning microscopy images of *E. coli* cells stained with LIVE/DEAD™ BacLight™ Bacterial Viability kit after treatment with distilled water (control), 200 mg/L NaOCl, air-MNB (30 min and 60 min), O_2_-MNB (30 and 60 min) and O_3_-MNB (30 and 60 min). Green cells indicate viable bacteria, red cells are dead bacteria. Scale bar = 200 px. (Color figure online)

After activating the cultures in LB, *E. coli and S. aureus* were transferred into centrifuge tubes, centrifuged at 8000×*g* to remove supernatant and resuspended in autoclaved distilled water. As shown in S-Fig. 2A, 1 mL of each bacterial cell suspension was added to 9 mL of respective treatments: (i) sterile distilled water (DW, as control) for 60 min, (ii) NaOCl (200 mg/L, for 5 min based on industry practice), while (iii) air-MNB, (iv) O_2_-MNB and (v) O_3_-MNB for 30 and 60 min. To mix and agitate the resultant mixture was stirred at 150 rpm using an orbital shaker at room temperature. To assess the effectiveness of air, O_2_ and O_3_ MNB at varying treatment times, cells of the bacterial (*E. coli* and *S. aureus*) interreacted for 30 and 60 min in a mixture with air, O_2_, and O_3_ MNB water. All treatments were conducted in triplicate (*n* = 3).

#### Confocal laser scanning microscope

The live and dead status of *E. coli and S. aureus* cells after treatment was investigated with confocal laser scanning microscopy (CLSM). For each treated samples ≈ 500 μL was taken and placed in a 2 mL Eppendorf tubes. It was stained with 0.5 μL of LIVE/DEAD™ BacLight™ Bacterial Viability kit (Invitrogen, Thermo Fisher Scientific, Eugene, Oregon) and 0.5 μL of propidium iodide in the dark. CLSM was performed with a Zeiss LSM780 with ELYRA PS1 platforms light microscope. The CLSM is equipped with an on-stage incubator for CO_2_, humidity and temperature control for live cell imaging, super-resolution platforms, and Argon laser with 3 excitation lines (458, 488 and 514 nm). In addition, cell viability was calculated by dividing the quantity of viable cells (green) on the image after a predetermined retention time by the count of viable cells (green) particles before retention in MNB water using Fiji ImageJ software, and the value was expressed as percentage survival rate (%), as earlier illustrated by Ferreira and Rasband (2012). The viable bacterial cells (%) were calculated from 3 random points on the CLSM images.

#### Scanning-transmission electron microscope analysis

To study ultrastructural changes of bacterial cell (*E. coli* and *S. aureus*) envelope after exposure to MNB or NaOCl, scanning-transmission electron microscope (STEM) analysis was conducted. Briefly, 1.8 μL of cell pellets was obtained from 500 μL of DW, NaOCl or MNB treated cell suspensions and fixed in EM fixative (2.5% glutaraldehyde, 4% formaldehyde in 0.1 M PB (with pH 7.4)) for 1 h at 4 °C. Thereafter, the samples were rinsed/washed for 5 min (3 times) in 0.1 M PB, and then incubated in 2% osmium for 1 h at 4 °C in a sealed container, followed by incubation with 1% uranyl acetate at 4 °C overnight. Subsequently, the samples were washed 3 times with DW between each incubation step. They were then dehydrated using ethanol stock concentrations of 20, 50, 70, 90 and 100%, and each for 10 min at 4 °C, then 100% dry ethanol and 100% acetone for 10 min. Sequential resin infiltration was assayed by diluting EPON resin in acetone and incubating in sequence of (1) 50: 50 acetone: EPON for 2 h (2) 25: 75 acetone: EPON for 2 h and (3) 100% EPON overnight.

Subsequent day, the resin was replaced with fresh 100% EPON, and the samples were placed in the oven in a sealed container to bake for 48 h at 60 °C. After “resin embedding”, the resin block was clipped to the region of interest using a Lecia UC7 ultramicrotome system (Leica Microsystems, Austria) with a 45° diatome (diamond knife). Clipped/trimmed sections were 100 nm thin, and the STEM grids containing the sections were placed into Apreo Volumescope (ThermoFisher, Netherlands). Xt Microscopy (Thermofisher) software was used to acquire the STEM micrograoh.

### Bacterial inactivation on guava fruit surface

Fresh ‘Fan Retief’ guava fruit were harvested from Modderkloof Boerdery in Paarl, Western Cape, South Africa, and transported to Agricultural Research Council-Infruitec Postharvest Pathology Laboratory and stored at 10 °C until use. Healthy guava fruit with uniform size (≈ 6 cm, diameter), weight (≈ 170 g), total soluble solid (11.2 ± 0.40 Brix) and titratable acid (0.7 ± 0.02) were used in this experiment. As shown in S-Fig. 2B, guava fruits were sanitized by spraying with 70% ethanol, followed by 5 min rinsing in sterile distilled water to remove native dirt and microorganisms. Then the fruit were air-dried in a biosafety laminar flow for 1 h. Thereafter, whole guava fruit were immersed in 500 mL inoculum suspension of *E. coli* and *S. aureus* for 30 min, then drained and air dried in a laminar flow biosafety cabinet for 1 h to promote attachment before washing with DW, NaOCl and the two most effective MNB feed gases from in-vitro bacterial inactivation study. One inoculated guava fruit was transferred into a beaker containing 1000 mL each of DW, NaOCl, air-MNB and O_3_-MNB. The beakers were placed on an rotary shaker (150 rmp) to simulate pack-line washing/decontamination for 60 min for DW, air- and O_3_-MNB, and 5 min for NaOCl at room temperature.

The microbial load of *E. coli* and *S. aureus* was evaluated by total plate count method before and immediately after dipping in solutions, and after storage at 13 °C for 12 days. This was carried out by removing surface microbes on inoculated guava by placing in 1000 mL beaker containing sterile physiological saline solution (PSS) and shaking on orbital shaker for 60 min. Thereafter, 1 mL was transferred from each beaker into 9 mL of sterile PSS for the threefold serial dilution step. Afterwards, 1 mL from each dilution was transferred into PCA plates, and incubated for 48 h at 37 °C. At the end of incubation, the colony forming units (CFU) were counted (25–250/plate) and transformed to log CFU/cm^2^. This was conducted in triplicate per dilution (*n* = 9).

### Statistical analysis

Statistical tests were analyed using GenStat 2015, 18.1 VSN International Ltd statistical software. Data was subjected to a two-way analysis of variance to investigate the main effects (treatments and storage duration), and their interaction. Differences between means were tested using Duncan Multiple Range Test at p ≤ 0.05. The results mentioned in this study are presented as mean ± SD.

## Results and discussion

### Size distribution and concentration of micro-nano bubble waters

The size distribution varied significantly with feed gases, and average diameter for air, O_2_ and O_3_ MNB were 83.0 ± 7.0 nm, 137.6 ± 11.7 nm, and 172.8 ± 13.8 nm, respectively. The highest MNB concentration were 1.29 × 10^8^ ± 4.56 × 10^7^ particles/mL for air MNB, followed by 7.36 × 10^7^ ± 6.66 × 10^6^ particles/mL for O_2_ MNB (Fig. 1). Moreover, SD is the measure of the width (spread) of the size distribution profile of bubbles. The SD of air MNB was found to be 63.1 ± 6.9 nm whereas that of O_2_ MNB was 80.6 ± 13.2 nm. Furthermore, D10, D50 and D90 values indicates the percentage of bubbles under size. For example, 10% of air MNB are 32.9 ± 6.2 nm or smaller giving another indication of spread of particles within the sample. Thus, 50 and 90% of air MNB were 61.1 ± 6.7 and 189.6 ± 29.8 nm or smaller, respectively. For O_2_-MNB, D10, D50 AND D90 were 49.4 ± 10.4, 115.6 ± 18.6, and 237.62 ± 0.3, respectively.

In general, air MNB had smallest bubble size and highest concentration compared with O_2_ MNB. The smaller diameter of air-MNB will benefit the bubbles residence time in solution according to Stokes’ Law, which states that bubbles with smaller diameter, has slower rise velocity to the surface (Saboni et al., 2016). Finally, due to a high internal Laplace pressure of MNB with smaller diameter (Uchida et al., 2016; Fan et al., 2019), the gas dissolution rate is higher which could have contributed to a higher air-MNB concentration than O_2_-MNB.

### Antimicrobial effects against *E. coli* and *S. aureus*

#### Confocal laser scanning microscope (CLSM)

**Fig. 3:**
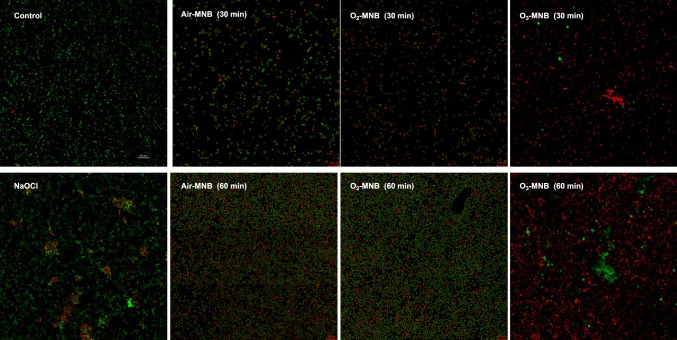
Confocal laser scanning microscopy images of *S. aureus* stained with LIVE/DEAD™ BacLight™ Bacterial Viability kit after treatment with distilled water (control), 200 mg/L NaOCl, air-MNB (30 min and 60 min), O_2_-MNB (30 and 60 min) and O_3_-MNB (30 and 60 min). Green cells indicate viable bacteria, red cells are dead bacteria. Scale bar = 200 px. (Color figure online)

**Fig. 4:**
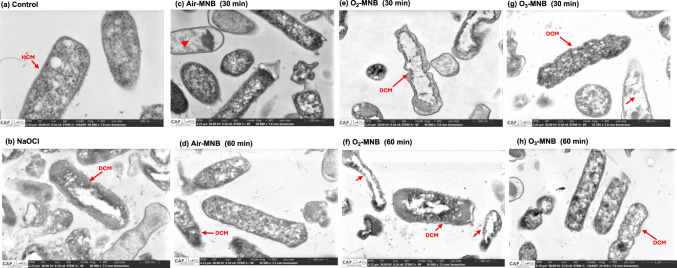
STEM micrographs of *E. coli* treated with distilled water (control), 200 mg/L NaOCl and MNB waters, red triangle and arrows indicates cell membrane detachment or loss of intracellular contents, *HCM* healthy cytoplasmic membrane, *DCM* damaged cytoplasmic membrane. (**A**) Control (cells have uniform cytoplasmic density, intact membrane), (**B**) 200 mg/L NaOCl (translucent cytoplasm, shrunken and misshapen cells, loss of turgidity, unhealthy membrane), (**C**) air MNB-30 min (some cells are electron sparse, islands of condensed chromatin), (**D**) air MNB-60 min (electron sparse cells, some cells have translucent cytoplasm), (**E**) O_2_ MNB-30 min (irregular and misshapen cell membrane, loss of intracellular contents), (**F**) O_2_ MNB-60 min (misshapen cell wall, cell at late stage of death, condensed chromatin), (**G, H** O_3_ MNB 30 and 60 min (some cells at late stage of death, condensed chromatin, detachment of cell membrane from cell wall, electron sparse cytoplasm, loss of turgidity, misshapen cells with loss of structure, cells with no cytoplasmic content. Bars represent scale of varying sizes. *When *E. coli* experiences stress, its shape could become significantly elongated and in other instances become spherical. Stressed *E. coli* can exhibit changes in cell wall thickness and periplasmic density. (Color figure online)

*Escherichia coli* and *S. aureus* were stained with LIVE/DEAD™ BacLight™ Bacterial Viability kit to monitor the viability of bacterial populations. Unhealthy and compromised bacterial cells membrane that are dead will stain red, whereas intact membrane and viable healthy bacterial cells will stain green. The bactericidal efficacy against *E. coli* was more pronounced under NaOCl and O_3_-MNB-60 min treatments, which recorded a cell viability of 5.97 and 6.73%, respectively (Fig. 2). Nevertheless, according to the CLSM images, air MNB-30 min, air MNB-60 min, and O_2_-MNB-30 min treatments had minimal impact on *S. aureus* population (Fig. 3). Results further demonstrated that air-MNB 60 min and O_2_-MNB 60 min were more effective than distilled water (control) in inactivating *E. coli* cells (S-Fig. 4A). This suggested that *S. aureus* was more resistant to air- and O_2_-MNB treatments as shown in S-Fig. 4B. The differences in sensitivity of tested microorganisms to MNB could be explained by the dissimilarity in the cell wall composition of the bacteria.

Previous investigations have also shown that MNB generated using absolute O_2_ was more effective against biofilm formation in comparison to MNB generated using air or pure CO_2_ (Rafeeq et al., 2020). Similarly, O_2_-MNB completely removed *Vibrio parahaemolyticus* biofilm on plastic coupons after 2 min exposure (Shiroodi et al., 2021). In the same study, O_2_-MNB caused up to 3 log decline of *E. coli* and *L. innocua* biofilms by attaching to the surface and reducing the surface tension and bacterial adhesion. However, based on our findings, the bactericidal impact of MNB was influenced by the feed gas types and were a better alternative sanitizer to NaOCl. Similar findings were reported by Hayakumo et al. (2014) who found that by using in-vitro time-kill assays with O_3_-MNB for 0.5 min led to < 10 CFU/mL of *Porphyromonas gingivalis* and *Aggregatibacter actinomycetemcomitans*. In another study, O_3_-MNB inhibited the growth of *E. coli* to < 3.3 log_10_ CFU/mL (Saijai et al., 2019). Recently, Gohari et al. (2024) showed that O_3_-MNB achieved 100% inactivation in *P. aeruginosa* and *S. aureus* at 5 g/h ozone, after 8 and 5 min, respectively.

The integrity of the *S. aureus* cell membrane affecting the support of the cell wall is that it is gram positive, and has a thicker and compact supported layer of peptidoglycans shielding against stress (Liu et al., 2022; Tang et al., 2017). Additionally, microbes have the capability to tolerate mild induced stressses such as exposure to MNB via the synthesis of ROS scavenging enzymes, release of stress-response proteins, and cell membrane repair (Tan et al., 2022). Nonetheless, *S. aureus* was not resistant to O_3_-MNB-60 min, which led to lower viable cells (54.46%) in comparison to the control and NaOCl (S-Fig. 4B). In addition, nearly over 50% of *S. aureus* cells treated with O_3_-MNB-60 min stained red (Fig. 3), indicating cytoplasmic membrane of most treated cells was injured. In the case of *E. coli*, the inactivation efficacy of NaOCl treatment was better that O_3_-MNB-30 min (p < 0.05), but similar to O_3_-MNB-60 min (p > 0.05), as shown in S-Fig. 4A. In this instance, the conventional NaOCl treatment was more effective than the control and other types of MNB treatments except for O_3_-MNB-60 min. However, as opposed to NaOCl, which produces toxic disinfectant by-products, and could deposit carcinogenic residue of ready to eat produce (Nyamende et al., 2023). These MNB treatments presents a green prospective for microbial inactivation. Similarly, MNB do not cause changes in flavour, taste, and scent of fruits and vegetables (Malahlela et al., 2024).

Furthermore, compared to the conventional NaOCl treatment to which several bacteria have developed multiple resistant mechanisms (Fabrizio et al., 2024). O_3_-MNB has a broad spectrum as an antimicrobial agent, and different microorganism species have inherent sensitivity to the gas (Unger et al., 2022). The inactivation of the microbes with O_3_ is a systemic process that occurs due to the damage of cellular envelope and subsequent dispersion of the intracellular constituents, since the gas has a high oxidative potential. Furthermore, O_3_ can to react with intracellular constituents such as proteins, unsaturated fatty acids, enzymes cell wall peptidoglycans, and nucleic acids. In aqueous media, such as O_3_-MNB, the inactivation of microorganism could be attributed to the O_3_ molecular and the superoxide, hydroxyl and the hydroperoxyl radicals, generated from the collapse of MNB (Malahlela et al., 2024).

In addition, pH and ORP are considered important factors affecting inactivation of microorganisms. Our current results demonstrated that O_3_-MNB treatments which had a low pH (2.71 ± 0.01) and high ORP (919 ± 12.28 mV) were effective in inactivating *E. coli* and *S. aureus* than control, air and O_2_ MNB treatments. These findings were consistent with Jhunkeaw et al. (2021) results, suggesting that high value of ORP in O_3_-MNB may offer a high microbial inactivation. Moreover, although O_3_-MNB had a bigger average size (172.8 ± 13.8 nm) than air (83.0 ± 7.0 nm) or O_2_-MNB (137.6 ± 11.7), this size could have been sufficient in increasing the surface area and mass transfer of O_3_ molecule in water, and increasing the oxidizing capacity of MNB for improved disinfection.

#### Scanning-transmission electron microscope analysis

**Fig. 5:**
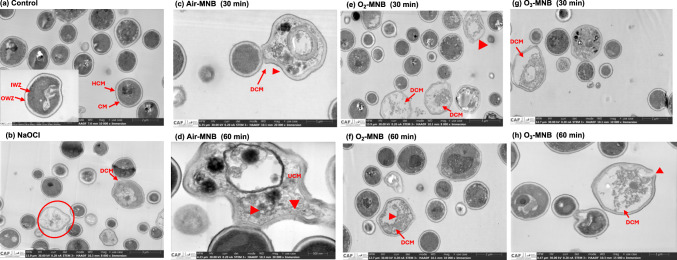
STEM micrographs of *S. aureus* treated with distilled water (control), 200 mg/L NaOCl and MNB waters. *IWZ* inner wall zone, *OWZ* outer wall zone, *CM* cytoplasmic membrane, *HCM* healthy cytoplasmic membrane, *DCM* damaged cytoplasmic membrane, red triangle indicates cell membrane detachment and loss of intracellular contents. (**A**) control (normal morphology, intact cell membrane and healthy cytoplasmic membrane), (**B**) 200 mg/L NaOCl (translucent cytoplasm, release of intracellular components, mishappen cells), (**C**) air MNB-30 min (some cells with no cytoplasmic content, intact cell membrane), (**D**) air MNB-60 min (detached cell membrane from cell wall, condensed chromatin, misshapen and irregular cell structure), (**E**) O_2_ MNB-30 min (some cells at late stage of death with loss of intracellular structures, condensed chromatin, cells with no cytoplasmic content), (**F**) O_2_ MNB-60 min (condensed chromatin, loss of intracellular structures), (**G**) O_3_ MNB-30 min (translucent cytoplasm, damaged membrane, cells with no intracellular structures, totally deformed cell structure), (**H**) O_3_ MNB-60 min (release of intracellular structures, mishappen cells with loss of structure).Bars represent scale of varying sizes. (Color figure online)

Morphological and ultrastructural changes in the bacteria samples were visually detected using STEM. Scanning-transmission electron microscope micrographs of *E. coli* and *S. aureus* bacteria’s after exposure to distilled water, NaOCl, air, O_2_ and O_3_ MNB are presented in Figs. 4 and 5, respectively. In control Fig. 4A, *E. coli* had even intracellular organization and intact cytoplasm integrity, no changes found in outer and inner wall zone, cytoplasmic membrane and periplasm region, indicating that the cell membrane was not damaged. Moreover, the DNA region appeared normal. In contrast, ultrastructural examination of *E. coli* by STEM revealed that majority of *E. coli* cells exposed to NaOCl and MNB sanitizers were collapsed and destroyed. For an instance, the morphology of *E. coli* cell under NaOCl (Fig. 4B) showed obvious shrunken and mishappen shape, loss of turbidity and wavy surface and dissolution of internal contents. Air-MNB for 30 and 60 min resulted to enlarged periplasm region and translucent cytoplasm (Fig. 4C, D). Moreover, O_2_-MNB for 30 min exposure resulted to condened chromatin (Fig. 4E). On the other hand, *E. coli* cells treated with O_2_-MNB-60 showed unhealthy cell membrane with cells at late stage of death (Fig. 4F). In addition, periplasmic space was filled with electron-dense materials. After treatment with O_3_-MNB for 30 and 60 min, some completely lysed cells are observed, intracellular/DNA structures disappear, detachment of cell membrane from cell wall, loss of cell structure with no cytoplasmic contents (Fig. 4g, h). Similar ultrastructural changes were reported on gram negative *Aeromonas veronii* bacterial surface after treatment with O_3_-nano-bubbles for 10 min (Jhunkeaw et al., 2021).

For *S. aureus* cells treated with distilled water was free of structural damages (inner wall zone, outer wall one and cytoplasmic membrane), suggesting that cells were healthy (Fig. 5A). However, cells treated with NaOCl were shrunken and showed loss of internal constituents (Fig. 5B), whereas air MNB-30 min (Fig. 5C) led to some intact cell membrane structures with few cells with no cytoplasmic contents suggesting leakage of intracellular compounds such as proteins and DNA. Detachment of cell membrane from cell wall and irregular cell structure was observed under air-MNB-60-min (Fig. 5D). O_2_-MNB for 30 min showed irregular distribution of interior organelles, condensed chromatin and loss on internal constituents (Fig. 5E). Furthermore, after 60 min exposure to O_2_-MNB, some cells had translucent cytoplasm (Fig. 5F).

Similarly, O_3_-MNB treatments for 30 and 60 min resulted to similar structural changes, which were more pronounced compared with other treatment groups (Fig. 5G, H). Ozone-MNB treated cells showed no cytoplasmic contents with mishappen irregular shape. This suggested a sub-lethal cellular damage to structures that caused complete cell lysis. In this study, the cells of *E. coli* and *S. aureus* were severly destroyed and their shape was deformed substantially by NaOCl and O_3_-MNB treatments which confirmns the CLSM results. Fan et al. (2021) also reported similar changes on *Bacillus subtilis* strain CMCC63501 treated with Ag/TiO_2_ photochemical combined with MNB. This effect was reported for the oxidative destruction of *Colletotrichum gloeosporioides* cells by Malahlela et al. (2025). Cell debris and internal disintegration were identified after the treatment, indicating that the cells were considerably damaged. Moreover, in Figs. 4 and 5, there appears to be other damaged microorganisms which could be due to the use of rainwater for generating MNB. Therefore, this present a limitation in assessing the antimicrobial efficacy of MNB against *E. coli* and *S. aureus.* In addition, in this study, scavenging experiments were not conducted to identify reactive nitrogen–oxygen species that are responsible for microbial inactivation. Nonetheless, our results demonstrated that MNB can cause cell membrane damage and it could be hypothesized that MNB reactive nitrogen–oxygen species (RNOS) from air, O_2_ and O_3_ (e.g. H_2_O_2_, ^·^OH, ^·^NO) were the primary active species causing any sub-lethal damage to the microorganisms as depicted in the STEM results. Moreover, in water the redox potential of ^·^OH is high (2.8 eV) but it is unstable, whereas H_2_O_2_ is weak (1.78 eV), but has a longer half-life of several days (Clark et al., 2010). This could be the cytoplasmic membrane permeation advantage of the active species. Hence, the results obtained supports the theory that generated RNOS species after MNB collapse, could play a role in inactivating the tested microorganisms, by first penetrating the cells prior to damaging the interior structures (Becton et al., 2017). In addition, other RNOS could be engaged in degrading the protective cellular/spore membrane, and once the cell lysis occurs disrupt of structural integrity could continue. Furthermore, MNB can cause bacterial cell membrane damage, protein oxidation and DNA decomposition (Shiroodi et al., 2021). Therefore, the proposed mechanism in which MNB inactivate microorganisms involves (a) collapsed MNB generating RNOS (b) RNOS attack cell membrane (c) permeation of RNOS to the interior of the cell (d) disruption of internal structures (d) breakage of cell wall and cytoplasmic membrane (e) leakage and disappearance of protein region and subsequent cell lysis.

### Bacterial inactivation on guava fruit surface

**Fig. 6:**
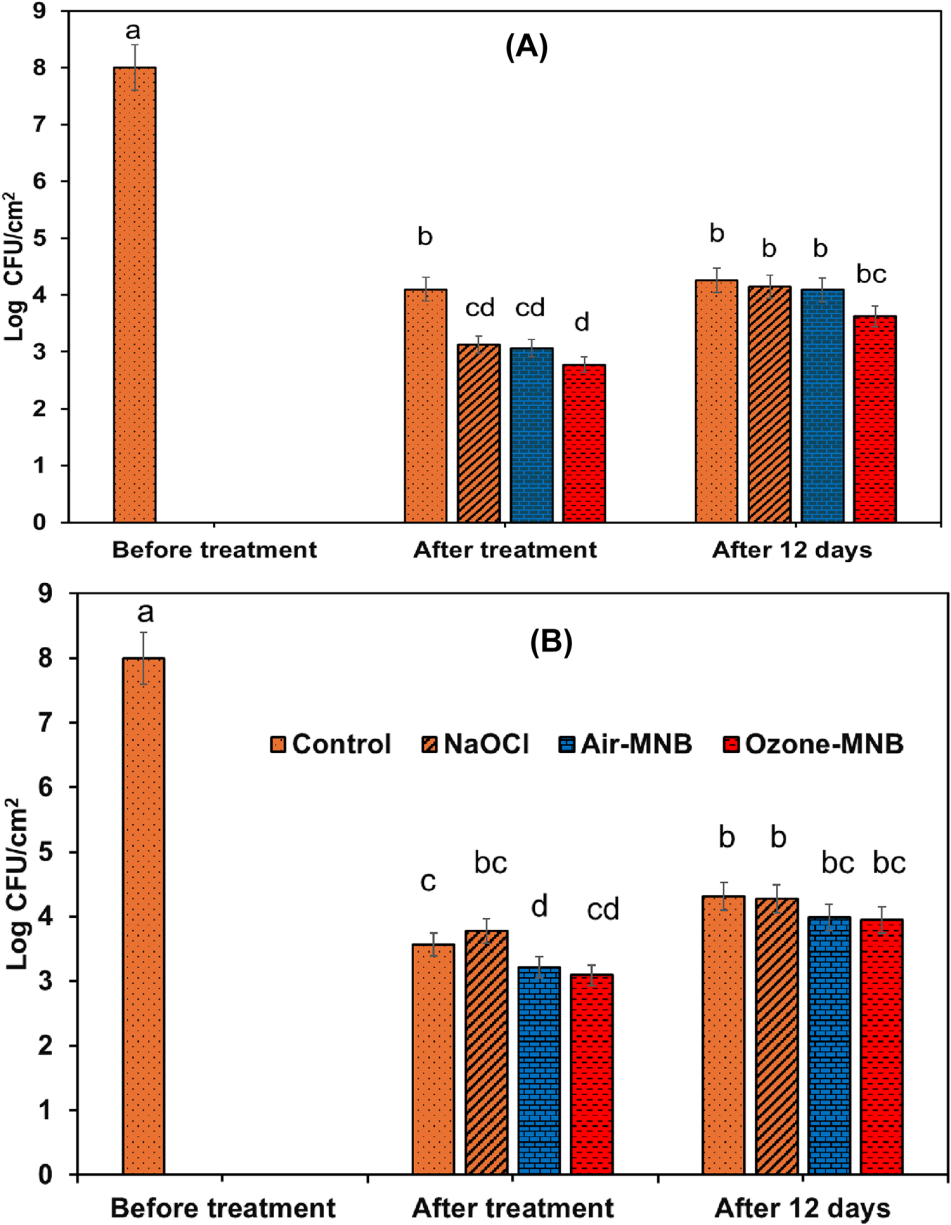
Bacterial counts for (**A**) *E. coli* and (**B**) *S. aureus* on ‘Fan Retief’ guava fruit surface after treatment with distilled water (control), 200 mg/L NaOCl, air-MNB (60 min) and O_3_-MNB (60 min) stored at 13 °C for 12 days. The bars indicate the standard deviation. Mean values denoted by similar letters are not significantly different at p < 0.05

The effect of MNB on removing bacteria from guava fruit surface was evaluated by soaking guavas contaminated with *E. coli* and *S. aureus* in distilled water (60 min), 200 mg/L NaOCl (5 min), and air and O_3_-MNB for 30 and 60 min, respectively at room temperature (Fig. 6A, B). The results illustrated that the interaction between MNB-feed gases and storage duration did not significantly (p > 0.05) change the *E. coli* and *S.aureus* bacterial count on guava fruit surface, while, storage duration had a significant influence (p ≤ 0.05). The initial count for *E. coli* (6.8 log CFU/cm^2^) and *S. aureus* (6.5 log CFU/cm^2^), declined immediately after treatment by > 3 log reduction across all treatment groups, but increased slightly after 12 days of storage (Fig. 6). These observations might be attributed to the decomposition of active antimicrobial compounds in NaOCl, air and O_3_-MNB or the recovery of bacterial cells during storage at 13 °C.

Notably, similar log reduction was recorded for O_3_-MNB and the conventionally used NaOCl immediately after treatment and 12 days of storage (Fig. 6). Ozone decomposition in water has great oxidizing capacity and plays an effective disinfection role, due to these advantages: its potency, lack of cytotoxicity or carcinogenicity effects, swift antimicrobial action, the ease of application as a dipping solution, and its biocompatibility compared with NaOCl (Goztas et al., 2014). Sodium hypochlorite is less biocompatible compared to aqueous-O_3_ for the human oral epithelial cells, periodontal cells and gingival fibroblast cells (D’Amario et al 2022; Goztas et al., 2014). In addition, the ingestion of ozonated drinking water poses no major risk, being that ozone has a very short half-life, and all the concentration present in water will decline to zero before getting to a consumer (Seridou and Kalogerakis, 2021). Furthermore, the dissolution of ozone in microbubbles have been to increase the solubility of ozone in water and the decomposition of residual organic compounds improved remarkably (Jabesa and Ghosh, 2016). Various studies have suggested possible correlations between the bubble size (diameter) and the improvement in ozone solubilization rate in solution. For example, Kobayashi et al. (2011) demonstrated that the concentration of aqueously dissolved ozone was higher when the water was treated with microbubbles compared to macrobubbles. Similarly, the dissolution of ozone under microbubbles and normal bubbles showed that the dissolved concentration was ≈ 2.5 times higher for microbubbles compared to normal bubbling (Takahashi et al., 2015). He et al. (2015) reported in their feasibility study that ozone dissolution via micro- and nanobubbles was approximately 50% higher after 5 min-aeration compared to conventional mixing pump with normal large bubbles. All these benefits make the application O_3_-MNB a suitable alternative to the current conventionally used NaOCl in the packhouse.

Incorporation of paracetic acetic acid with CO_2_-MNB solution also resulted in 4.4 log reduction (Singh et al., 2021). Moreover, Zhang and Tikekar (2021) reported that air MNB-assisted washing with 100 mg/L NaOCl lead to ≈ 3.3, ≈ 1.0, and ≈ 1.0 log reduction for the surface of grape tomatoes, blueberries, and baby spinach. Ozone-MNB water showed bactericidal influence on various gram-negative and -positive bacteria strains (Osman et al., 2020). Scavengers of ROS experiments reveleaved that H_2_O_2_ and ^·^OH ROS were the major active species of air-MNB for inhibition of *Bacillus subtilis* spores (Fan et al., 2021). The physicochemical characteristics of treatment solutions were also studied, including pH and ORP.

Ozone MNB generated the highest ORP (919 ± 12.28 mV), indicating stronger oxidizing power of bacterial components such as membrane proteins, metabolic enzymes and DNA. Subsequently disrupting intracellular processes, and resulting in effective antimicrobial efficacy (Moonsub et al., 2024). Nonetheless, there is a potential health risk through direct exposure to O_3_ gas inhilation due to its corrosiveness. More severe exposure to levels of 1.5 to 2 mg/L for 2 h can produce acute symptoms including chest pains (Seridou and Kalogerakis, 2021). Therefore, regardless of O_3_-MNB bactericidal effectiveness over air MNB; air MNB does not contain potential hazardous compounds and is less costly to operate (readily available from the atmosphere) which could be preferred by packhouses.

Overall, *S. aureus* was more resistant to the MNB treatments. Nevertheless, O_3_-MNB-60-min showed the potential as an alternative to NaOCl to inactivate *S. aureus*. These results indicates that the effectiveness of MNB treatments could impacted by the types microbes, and MNB system optimization would be crucial. The study further demonstrated that O_3_-MNB-60 min inhibited the growth of *E. coli* and *S.aureus* on the surface of guava fruit after day 12 of storage. Future research is needed to investigate the implication of repeated usage of MNB water batch on the efficacy. Similarly, focus on the characterization of free scavenging radicals and active species in MNB water systems will elucidate on the microbial inactivation mechanism.

## Supplementary Information

Below is the link to the electronic supplementary material.

Supplementary file1 (DOCX 1011 KB)

## Data Availability

Data of this study is available upon request.
