# Supplementary material for: Impacts of feed gases for micro-nano bubble water treatments: Antimicrobial efficacy against Escherichia coli and Staphylococcus aureus on ‘Fan Retief’ guava fruit
Source: Food Sci Biotechnol. 2025 May 20;34(12):2959–70. doi: 10.1007/s10068-025-01897-w (PMC12240903; doi:10.1007/s10068-025-01897-w)
Supplement: Supplementary file 1 — Supplementary file1 (DOCX 1011 KB) [file 10068_2025_1897_MOESM1_ESM.docx]

**Supplementary (S) Figures and Tables**

**S-Figure 1**. The conceptual model and schematic diagram for micro-nano bubble water generator system with different feed gases, and characterization of MNB physicochemical properties.

**S-Table 1.** Physico-chemical characteristics of treatments

| **Treatments** |  | **pH** |  | **ORP (mV)** |  | **Temperature (°C)** |
| --- | --- | --- | --- | --- | --- | --- |
| Distilled water |  | 6.45 ± 0.45 ^c^ |  | 286 ± 31.00 ^c^ |  | 18.1 ± 1.90 ^c^ |
| NaOCl |  | 10.74 ± 0.00 ^a^ |  | 478 ± 1.00 ^b^ |  | 21.6 ± 0.00 ^ab^ |
| Air-MNB |  | 9.50 ± 0.50 ^b^ |  | 188 ± 18.00 ^d^ |  | 19.3 ± 0.70 ^bc^ |
| O_2_-MNB |  | 9.32 ± 0.68 ^b^ |  | 232 ± 12.00 ^c^ |  | 19.7 ± 0.30 ^bc^ |
| O_3_-MNB |  | 2.71 ± 0.01^d^ |  | 919 ± 12.28 ^a^ |  | 21.3 ± 0.40 ^ab^ |
| Mean values (*n* = 5) ± standard deviation, and similar lower-case letters along the columns are not significantly different at p ≤ 0.05. | | | | | | |

**S-Figure 2**. Experimental layout for bacterial inactivation (**A**) in cell suspension and (**B**) on guava fruit surface stored at 13 °C for 12 days.


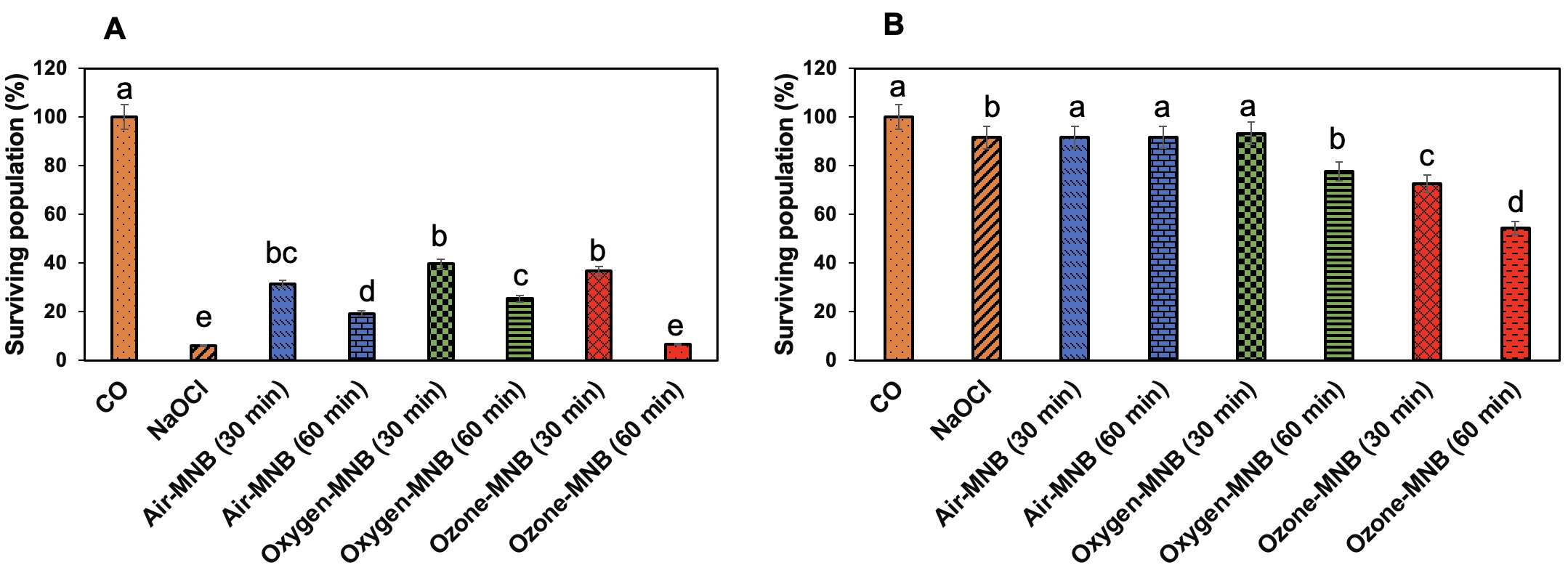


**S-Figure 4**. Survival or viable cells (%) of (**A**) *E. coli* and (**B**) *S. aureus* after treatment with distilled water (CO), NaOCl (200 mg/L), air-MNB (30 min and 60 min), O_2_-MNB (30 and 60 min) and O_3_-MNB (30 and 60 min). The error bars indicate the standard deviation. Mean values denoted by similar letters are not significantly different at p ≤ 0.05.
